# Supplementary material for: Increased Citrullinated Histone H3 Levels in the Early Post-Resuscitative Period Are Associated with Poor Neurologic Function in Cardiac Arrest Survivors—A Prospective Observational Study
Source: J Clin Med. 2019 Oct 1;8(10):1568. doi: 10.3390/jcm8101568 (PMC6832426; doi:10.3390/jcm8101568)
Supplement: Supplementary file 1 [file jcm-08-01568-s001.pdf]

**Table S1.** Logistic regression.

| | 30-day poor neurologic function | |
|---|---|---|
| | Odds ratio 95% confidence interval | *p*-value |
| **H3Cit 12h** | **1.6 (1.1-2.3)** | **0.029** |
| **H3Cit 12h** | 1.5 (0.9-2.2) | 0.064 |
| **Age** | 1.0 (0.9-1.1) | 0.159 |
| **H3Cit 12h** | 1.6 (1.0-2.3) | 0.028 |
| **Male Sex** | 1.7 (0.5-5.8) | 0.423 |
| **H3Cit 12h** | 1.6 (1.1-2.4) | 0.023 |
| **Location (home)** | 1.6 (0.5-5.1) | 0.385 |
| **H3Cit** | 1.5 (1.0-2.3) | 0.034 |
| **Witnessed** | 0.6 (0.1-3.8) | 0.584 |
| **H3Cit 12h** | 1.6 (1.0-2.3) | 0.029 |
| **Basic Life Support** | 0.8 (0.2-2.6) | 0.678 |
| **H3Cit 12h** | 2.0 (1.2-3.3) | 0.010 |
| **No-flow time** | 3.7 (1.2-12.0) | 0.023 |
| **H3Cit 12h** | 1.4 (0.9-2.2) | 0.092 |
| **Low-flow time** | 1.0 (0.9-1.0) | 0.535 |
| **H3Cit 12h** | 1.4 (0.9-2.2) | 0.101 |
| **Non-shockable** | 12.2 (1.4-107) | 0.024 |
| **H3Cit 12h** | 1.5 (1.0-2.3) | 0.043 |
| **Epinephrine** | 1.3 (0.9-1.7) | 0.070 |
| **H3Cit 12h** | 1.6 (1.0-2.4) | 0,035 |
| **Lactate** | 2.8 (0.9-7.8) | 0.055 |
| **H3Cit 12h** | 1.5 (1.0-2.3) | 0.048 |
| **D-dimer** | 1.9 (1.1-3.4) | 0.023 |

**Table S2.** Score test for trend.

| Biomarker level Quintiles | P value |
|---|---|
| nptrend **quin_hist_0h**, by(poor_outcome) | z = 0.18<br>Prob > \|z\| = 0.854 |
| nptrend **quin_hist_12h**, by(poor_outcome) | z = 2.22<br>Prob > \|z\| = 0.026 |
| nptrend **quin_cfdna_0h**, by(poor_outcome) | z = 2.77<br>Prob > \|z\| = 0.006 |
| nptrend **quin_cfdna_12h**, by(poor_outcome) | z = 2.22<br>Prob > \|z\| = 0.026 |
| nptrend **quin_nucleosome_0h**, by(poor_outcome) | z = 2.22<br>Prob > \|z\| = 0.027 |
| nptrend **quin_nucleosome_12h**, by(poor_outcome) | z = 2.42<br>Prob > \|z\| = 0.016 |

We used a score test to assess a trend of increasing biomarker levels at specific time points for neurologic outcome. The score test is a nonparametric test for a trend across ordered groups as an extension of the Wilcoxon rank-sum test. This has been added to the manuscript. An appropriate reference has been added: Cuzick, J. 1985. A Wilcoxon-type test for trend. Statistics in Medicine 4: 87-90.

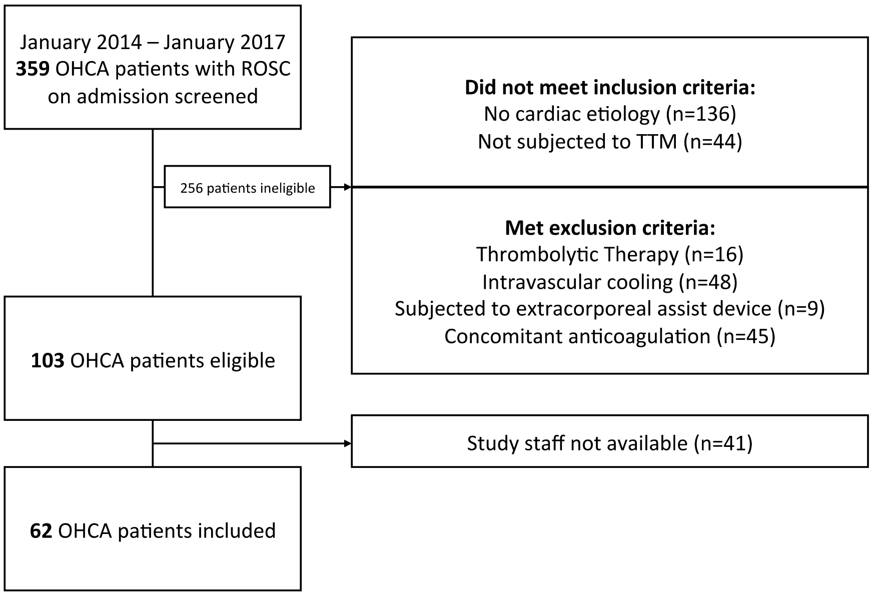


**Figure S1.** Study flow chart showing the selection process of study patients. Sum of n exclusions (298) exceeds the number of patients who were not eligible for study enrollment (n=256), due to fulfillment of more than one exclusion criterion. OHCA, out-of-hospital cardiac arrest; ROSC, return of spontaneous circulation; TTM, targeted temperature management.

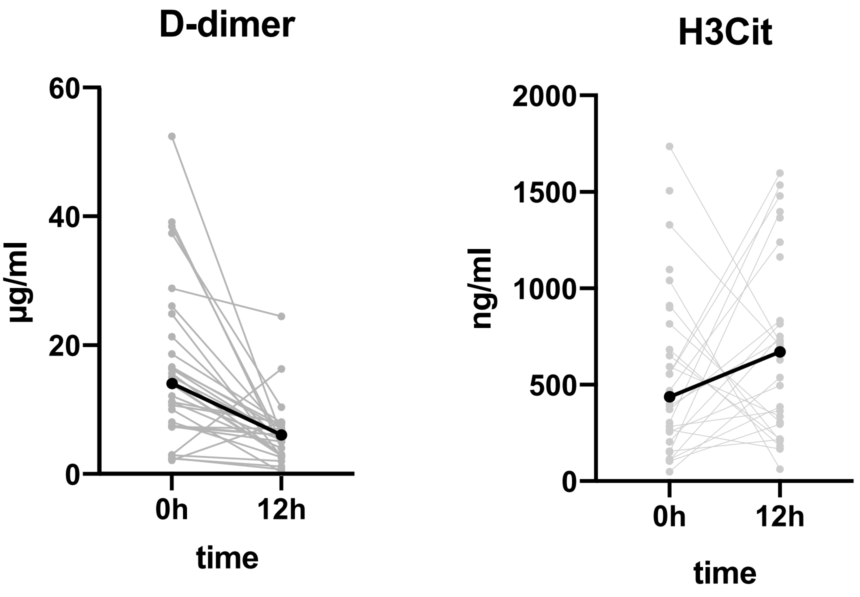


**Figure S2.** 0h and 12h plasma levels of D-dimer and H3Cit in patients with poor 30-day neurologic function. Similar to median cfDNA and nucleosome levels, median d-dimer levels decreased from admission to 12 hours. Grey lines indicate individual data points, black lines represent median marker levels. There was no correlation between 12h d-dimer and 12h H3Cit levels (rho=0.100, p=0.650).
